# When parents play favorites: brood demand shapes parental preference for offspring UV color

**DOI:** 10.1093/beheco/arad040

**Published:** 2023-05-18

**Authors:** Jorge García-Campa, Wendt Müller, Alicia Rodríguez-Juncá, Judith Morales

**Affiliations:** Department of Evolutionary Ecology, National Museum of Natural Sciences – Spanish National Research Council (CSIC), c/ José Gutiérrez Abascal 2, 28006 Madrid, Spain; Department of Biology, Behavioural Ecology and Ecophysiology Research Group, University of Antwerp, Universiteitsplein 1, 2610 Wilrijk, Belgium; BIOECOMAC, Department of Animal Biology, Edaphology and Geology, University of la Laguna, Tenerife, Canary Islands, Spain; Department of Evolutionary Ecology, National Museum of Natural Sciences – Spanish National Research Council (CSIC), c/ José Gutiérrez Abascal 2, 28006 Madrid, Spain

**Keywords:** offspring quality signals, parental care, parental favoritism, parent-offspring conflict, UV coloration

## Abstract

Parents might initially produce more offspring than they might be able to raise. However, when offspring demand exceeds their parents´ rearing capacity, parents might shift care towards the offspring which yield greater fitness returns to achieve their optimal brood size via brood reduction. Such favoritism could rely on offspring signaling traits if these inform parents about offspring quality and hence about the pay-offs of their investment. Here we investigated whether favoritism of blue tit (*Cyanistes caeruleus*) parents for an offspring signal (i.e., ultraviolet (UV) plumage coloration) varies with brood demand. To test this, we experimentally blocked the UV reflectance of yellow breast feathers in half of the nestlings of each brood, and then we sequentially performed two opposing brood size manipulations to vary nestling demand below or above parental rearing capacity. In reduced broods, nestlings begged overall less intensely and gained more body mass, supporting that parental rearing capacities sufficed to satisfy brood demand. Moreover, in reduced broods, UV-blocked nestlings (i.e., low-quality offspring) were fed and prey-tested more often. Yet, they begged more than control nestlings, suggesting that they were perhaps treated differently by other family members or which they may exploit parental preferences beyond actual need (at least in reduced nests). Parents flexibly shifted their feeding rate and favoritism in response to short-term changes in family size, as there was no parental preference for enlarged broods. Such flexible parental feeding rules may allow parents to gain the upper hand in parent-offspring conflict. However, we did not find evidence that parental favoritism facilitated brood reduction, at least in conditions where demand was temporally enhanced.

## Teaser

Blue tit parents may produce more offspring than they are able to raise, and they might therefore preferentially invest in high quality offspring that are most likely to survive. Yet when parents are able to raise all their offspring they might even favor those with lowest survival probabilities to maximize reproductive success. Parents hence benefit from flexible feeding strategies for which they rely on offspring signals (i.e., colorful traits), which inform about offspring quality.

## INTRODUCTION

Parental care increases offspring fitness, but it is costly and it compromises the survival probability and future reproductive success of the caregiver (reviewed by Alonso-Álvarez and Velando 2012). Therefore, to maximize fitness, parents must balance their resource allocation across breeding events, at least in species with multiple breeding opportunities (Stearns 1992). Furthermore, in species raising multiple offspring simultaneously, parents may also vary resource allocation within broods. When there is post-natal parental care, parents may for example hedge their bets and produce more young than they will be able to raise (Gustafsson and Sutherland 1988), as they cannot exactly predict their rearing capacity at the time that their offspring are born.

If rearing conditions are good, parents should favor the neediest young to maximize the number of viable offspring (e.g., Davis et al. 1999; Brode et al. 2021), thus after a brood survival strategy (Lack 1947; Pijanowski 1992). On the other hand, when rearing conditions are more limited, parents might skew their investment towards high-quality offspring with higher survival probability (Davis et al. 1999), thus after a brood reduction strategy (Haig 1990). Hence, whether parents favor certain offspring more than another within a given breeding event could be a flexible decision which depends on their parental rearing capacity (Hinde and Kilner 2007). Parents should strategically vary their resource allocation among offspring within a given breeding event (i.e., parental favoritism) (Ward et al. 2009; Naguib et al. 2010; Thorogood et al. 2011; Shizuka and Lyon 2013; Royle et al. 2014) because the distribution of parental care among offspring which are raised simultaneously should vary according to the reproductive value of the young, which might also depend on the environmental conditions at the time of breeding (Clutton-Brock 1991). Therefore, parental favoritism can occur whenever there are fitness benefits for parents of differential investment among offspring (e.g., in the context of sex allocation, Komdeur 2012; or within-brood variation in offspring age, Jeon 2008).

Yet, such parental favoritism requires that parents make informed decisions based on offspring quality and need (Wright and Leonard 2002; Hinde and Godfray 2011). In birds, a number of offspring traits have been identified to reliably signal quality and to guide parental favoritism, such as the ultraviolet (UV) coloration of skin, mouth ,and feathers (Jourdie et al. 2004; Bize et al. 2006; de Ayala et al. 2007). In addition, offspring actively beg for food through vocal and postural displays, which can be adjusted in the short-term in the function of need (Wright and Leonard 2002; Caro et al. 2016). Hence, parents can use a combination of conspicuous ornaments and begging behaviors to evaluate the need and quality of their young. Such parental favoritism may, however, increase sibling competition for access to parental resources, as the resources are not equally accessible to the offspring (Royle et al. 2012). However, when parental favoritism depends on environmental conditions of which offspring might not be aware, revealing information may not necessarily be in the benefit of the offspring, also because parental and offspring interests are not always aligned (Trivers 1974; Pettifor et al. 2001). It, therefore, remains to be shown whether revealing one’s own quality is adaptive from the offspring’s perspective (Agrawal et al. 2001; Estramil et al. 2017). Yet, we need to know how parental feeding decisions are guided by offspring signals of quality (e.g., parents favor the most ornamented nestlings based on plumage coloration in Lyon et al. 1994; Ligon and Hill 2010; or skin UV reflectance in Bize et al. 2006) and whether which varies in function of the parents’ rearing capacity.

To test this hypothesis, we used the blue tit (*Cyanistes caeruleus*) as a study model. In this species, the prominent yellow breast plumage is a carotenoid-based trait, which reflects light both in the human visible (yellow to red wavelengths; 550–700 nm) and in the ultraviolet region (300–400 nm) of the reflectance spectrum (Johnsen et al. 2003; Jacot and Kempenaers 2007). In adults, there is significant evidence that both the carotenoid-based coloration (del Cerro et al. 2010; García-Navas et al. 2012; Midamegbe et al. 2013) and the UV component of breast feathers (García-Campa et al. 2022) function as signals of quality. In nestlings, both the carotenoid-based and the UV component co-vary with body mass (Johnsen et al. 2003; Jacot and Kempenaers 2007; Morales and Velando 2018; see also Galván et al. 2008, in the closely related great tit, *Parus major*), and nestling UV chroma is reduced in experimentally enlarged broods, in which competition for food might be enhanced (Jacot and Kempenaers 2007). Interestingly, cavity-nesting passerines are better at detecting changes in UV reflectance than in visible reflectance (Hunt et al. 2003; Avilés et al., 2006; Wiebe and Slagsvold, 2009). Accordingly, in the blue tit, it has been shown that, in particular, the UV component mediates social interactions among siblings as well as between parents and offspring (Morales and Velando 2018; García-Campa et al. 2021). In addition, when resources are limited, parents favor nestlings with higher UV chroma, thus, presumably those with higher quality (Morales and Velando 2018; García-Campa et al. 2021).

This suggests that parents might use nestling (UV) coloration in their decisions more than brood reduction, which is common in this species, given that it has one of the largest clutch sizes for its body size (Stenning 2018). Yet, parental preferences should be flexible, as the benefits of favoring specific offspring (e.g., those signaling high quality) are supposed to depend on the parental capacity to raise all offspring. To experimentally test this possibility, we first blocked the UV reflectance of yellow breast feathers in half of the nestlings in all broods to reduce perceived individual quality. Second, we performed two sequential and opposite brood size manipulations (i.e., increased and reduced or reduced and increased size) on two consecutive days to vary nestling demand below or above parental rearing capacity. We predicted that blue tit parents should favor poor-quality offspring (UV-blocked nestlings) if their parental rearing capacity suffices for rearing the whole brood (reduced broods). However, parents should favor high-quality offspring (non-UV-blocked nestlings) if the brood size exceeds their rearing capacities (enlarged broods), ultimately leading to brood reduction.

## MATERIALS AND METHODS

### Ethical note

All the methods were performed by the Guidelines for the Treatment of Animals in Behavioural Research and Teaching from the Association for the Study of Animal Behaviour/Animal Behaviour Society (ASAB/ABS) (2012), and with the Spanish laws for animal research. The study was approved by the Consejería de Medio Ambiente, Administración Local y Ordenación del Territorio, Comunidad de Madrid (PROEX 237/17).

### General methods and experimental manipulations

The study was conducted during the spring of 2019 in a wild population of blue tits breeding in a deciduous forest in Miraflores de la Sierra, Madrid, Spain (40°48ʹN, 03°47ʹW). Blue tit pairs produce one clutch per breeding season and both parents contribute to raise the brood which may be up to 15 nestlings (average brood size in the study population: 9.6 ± 1.8 SD; *n* = 464; range 4–15; data from years 2011, 2017–2019, and 2021). Nest-boxes were checked regularly to determine the laying date, clutch size and hatching date (=day 0).

On day 11, when offspring plumage is mostly completely developed, we experimentally blocked the UV color of yellow breast plumage in half of the nestlings of the brood with a permanent marker (Edding 4500; code 005). We reduced the reflectance in the UV region (300–400 nm) to resemble low-quality individuals which express a low-quality signal (UV-blocked nestlings) (see *Offspring color manipulation* below). The other half of the brood was control-treated (non-UV-blocked nestlings). All nestlings were ringed and weighed before treatment. We also measured the original UV color of each nestling and collected 5–10 breast feathers per nestling for molecular sexing (see *Molecular sexing of nestlings* below).

On day 11 and after UV manipulation, we performed the first reciprocal brood size manipulation between two nests (=dyads) (see *Brood size manipulation* below). On the morning of day 12, we first recorded the behavior of blue tit families in the enlarged/reduced nests and then re-weighed all nestlings. We then performed the second reciprocal brood size manipulation where we alternated the type of brood manipulation within each dyad (i.e., the brood which was enlarged on day 11 was reduced on day 12, and the brood which was reduced on day 11 was enlarged on day 12). On the morning of day 13, we recorded the behavior in all nests again and then re-weighed all nestlings.

### Offspring color manipulation

On day 11 of nestling age, and before the manipulation of UV color of the yellow breast feathers, we measured the original UV color of each nestling using a portable spectrophotometer (JAZZ, Ocean Optics). Color was extracted using CLR program v 1.1 (Montgomerie 2009) and UV chroma was calculated as the reflectance in the UV range divided by the total reflectance in the avian visual range (*R*_320–400_/*R*_300–700_; adapted from Johnsen et al. 2003, 2005). Therefore, we ensured that UV-blocked and non-UV-blocked nestlings did not differ in UV chroma before treatment (*F*_1,399_ = 0.38; *P* = 0.54). Half of the nestlings of the brood were UV-blocked using a yellow marker (Edding 4500, code 005) (Galván et al. 2008; Morales and Velando 2018). As a result of this manipulation, UV-blocked nestlings show lower reflectance in the UV region (300–400 nm) (García-Campa et al. 2021), as typically expressed by individuals in lower conditions. To control for possible side-effects of the permanent marker, the other half of the brood was control-treated using the same marker on a similar-sized region of inner primary feathers. This is the best candidate body part of nestlings which cannot be perceived by other family members. Parents (or siblings) cannot see the nestlings’ underwing plumage during their usual activities in the nest, like feeding or arranging the nest material, even when nestlings flap their wings (J. García-Campa and J. Morales, personal observations during many years of analyzing videos; e.g., Morales and Velando 2018; García-Campa et al. 2021; see also Galván et al. 2008 in great tits). We first randomly assigned the non-UV-blocked or the UV-blocked treatment to the first nestling on the offspring size hierarchy in the first experimental nest. Then, we subsequently alternated treatments between the after nestlings along the size hierarchy when processing the rest of the brood. We then alternated the non-UV-blocked and the UV-blocked treatment to the first nestling in the subsequent nest. Nestlings from the UV-blocked and non-UV-blocked treatments did not differ in body mass before treatment (*F*_1,401_ = 0.21; *P* = 0.65). Moreover, we also marked nestlings on the right or left side of the head according to their UV treatment using a white marker (Edding 751, code 049). Thus, nestlings’ treatments could be distinguished during the behavioral analysis (although the observer was “blind” according to the treatment; see *Video recordings and behavioral analyses*). White markings on the head were probably visible for the parents, but in previous experiments, parents responded to the offspring UV color regardless of whether offspring’s had a white mark or not (Morales and Velando 2018; García-Campa et al. 2022). Moreover, parental feeding rates and nestling begging intensity do not differ according to the side of the head on which white markings are placed (both *P* > 0.8; data from 2017; *n* = 95 nestlings).

### Brood size manipulation

We manipulated the brood size as such which each nest was subjected to two short-term manipulations of its original family size: (1) enlarged brood size, via an addition of two nestlings to the original brood size, and (2) reduced brood size, via the removal of two nestlings. First, on day 11, we randomly assigned the order of these manipulations so that within a dyad (=a pair of nests) one nest was enlarged and one nest was reduced. On the following day, each nest within a dyad received the opposite brood size manipulation than on the previous day. We exchanged nestlings between pairs of nests with similar brood size (±2 nestlings) and hatching date (±1 day). On day 11, the first family size manipulation was performed as follows: in each dyad (for example, nests *X* and *Y*) all original nestlings from nest *X* were marked with white paint (the same used for the head) on the right scapular region and all nestling from nest *Y* on the left one. Thus, it was possible to ascertain the original nest of each nestling after the exchange. To increase the family size in nest *X*, four nestlings from nest *Y* were transferred to *X*, and simultaneously, two nestlings from *X* were transferred to *Y*. This exchange ensured that both nests received chicks from a foreign nest. In each exchange, the same number of UV-blocked and non-UV-blocked nestlings were transferred. On day 12, the second exchange consisted in transferring four nestlings from nest *X* to nest *Y*, being two of them originally from nest *Y* and two from nest *X*, and ensuring a similar number of UV-blocked and non-UV-blocked chicks. Thus, both nests again contained foreign chicks. After this second exchange, brood *X* was reduced by two nestlings brood *Y* was enlarged by two. Finally, after the second video recording, we returned all foreign chicks to their original nest. To maintain the offspring size hierarchy between nestlings, we exchanged nestlings from medium positions in the size hierarchy in both nestling exchanges. The exchange of nestlings occurred early in the morning. On average it was finalized at 12:30 AM ± 1.5 h (mean ± SD). The following day, the video recordings started on average at 10:45 AM ± 0.5 h (mean ± SD). Therefore, nestlings were approximately 24 h in the experimental nest before they were recorded.

We expected that family members quickly adjust their behavior in response to the experimental manipulations, as parents are known to respond in a very short term to changes in brood demand, which can be induced by brood size manipulations, partner removal experiments, or studies in which parents are subjected to the playback of begging calls, with quasi immediate effects on parental provisioning behavior, both in this (García-Navas and Sanz 2010; Lucass et al. 2016; Griffioen et al. 2019; Iserbyt et al. 2019) and other species (e.g., house wrens *Troglodytes troglodytes*: Bowers et al., 2014; great tits *Parus major*: Hinde 2006; Hinde and Kilner, 2007; common starling *Sturnus vulgaris*: Wright and Cuthill 1990). Nestlings also respond in the short-term to these changes by adjusting their begging intensity (e.g., barn owl *Tyto alba*: Dreiss et al. 2015, 2017), which can also affect their current condition (see Bize et al. 2006 in the alpine swift *Tachymarptis melba* and the European starling *S. vulgaris*; see Morales and Velando 2018; García-Campa et al. 2021, in the blue tit).

### Video recordings and behavioral analyses

On day 11, we substituted the original nest-box of each nest with a recording nest-box to familiarize blue tit pairs with it before video recordings. The recording nest-box had the same size and features as the original one but with an opening in the ceiling (a round hole of around 8 cm in diameter) on which we placed a fake camera and an opaque plastic cover on it. Parents typically resume their feeding behavior soon after replacement, many times even when the recording nest-box is being hanged in the tree branch. On the morning of days 12 and 13, we substituted the fake camera with a real night-vision camera (DX, 8 LED and 180^o^ angle, China). We initially included 46 nests in the experiment but could only record parental provisioning in 40 nests, because in six nests the recordings failed. In addition, we could analyze the behavior of blue tit families both in enlarged and reduced broods in 39 nests. However, due to recording problems in one nest, we could only record the behavior when the brood was reduced. We observed the behavior of all family members for exactly 30 min. The first half an hour and the last 10 min of the video recording were excluded to avoid possible disturbance effects due to the researcher’s presence after placing the camera and manipulating the nest. A single observer analyzed all video recordings and was unaware of how the white marks had been assigned according to UV treatment (i.e., UV-blocked and non-UV-blocked treatment). During each parental provisioning event, we registered the following variables for UV and non-UV-blocked chicks: (i) the feeding rate (i.e., the number of prey items received); (ii) the begging intensity directed to the provisioning parent; and (iii) the number of nestlings which were prey-tested by the provisioning parent. Prey-testings—or prey withdrawal—occur during parental feedings, when parents place a prey item into a nestling’s gape but remove it before the nestling can swallow it. This behavior occurs frequently in this species and has been interpreted both as a gape size constraint (Wiebe and Slagsvold 2012) and as a “hunger test” which parents use to assess offspring needs (García-Campa et al. 2021), which imposes a cost to the offspring in terms of body mass gain (Morales and Velando 2018). Begging intensity was recorded after a 5-point-scale (0 = calm, 1 = weak gaping, 2 = gaping and neck stretched, 3 = gaping, neck stretched, and standing, 4 = gaping, neck stretched, standing, and wing flapping; Morales and Velando 2018; García-Campa et al. 2021). We then calculated the following values for non-UV-blocked and UV-blocked nestlings: (i) the feeding rate (i.e., the total number of prey items which the nestlings received according to their UV treatment); (ii) the mean nestling begging intensity per nest according to their UV treatment; and (iii) the mean number of nestlings which were prey-tested by parents during a feeding bout, again according to their UV treatment. For each nestling, we calculated body mass change twice (i.e., body mass on day 13 minus body mass on day 12, and body mass on day 12 minus body mass on day 11) to obtain a measure for the enlarged and the reduced brood condition. We obtained the body mass change of nestlings in 46 nests.

### Molecular sexing of nestlings

DNA was extracted from 25 mg of feather sheaths using the Qiagen DNeasy Blood and Tissue kit (Qiagen Inc, Valencia, CA, United States of America). Sex identification was performed by polymerase chain reaction (PCR) amplification of the CHD-W and CHD-Z genes with primers P2 and P8, following Griffiths et al. (1998) with a few modifications. An initial denaturizing step at 94°C for 4 min 30 s was followed by 40 cycles of 94°C for 30 s, 49°C for 45 s and 72°C for 45 s. A final run of 72°C for 10 min completed the program. Amplification was carried out in a total volume of 10 µl. Each PCR sample contained: 2 µl DNA, 0.08 µl Taq polymerase (Takara Bio Inc, Japan), 0.8 µl dNTP 2.5 mM, 0.5 µl of each primer 10 µM, 1 µl of 10× PCR buffer and 5 µl of sterilized distilled water. The sex of 12 chicks from five nests could not be determined due to unsuccessful DNA extraction.

### Statistical analyses

All analyses were performed with SAS 9.4. (SAS Inst., Cary, NC, United States of America), using the mean values per treatment in each nest. To analyze feeding rates, we performed a generalized linear mixed model with Poisson distribution, whereas the mean number of nestlings which were prey-tested and mean begging intensity were analyzed with mixed models with normal distribution. The mean number of nestlings which were prey-tested was “log_10_ (*x*+1)” transformed to fulfill the assumption of normality. For the three behavioral variables, we had four data per nest corresponding to non-UV-blocked and UV-blocked chicks in both the enlarged and the reduced brood size treatment. All models included dyad ID and nest ID (nested within dyad) as random factors, and two random slopes (nest ID × UV treatment and nest ID × brood-size treatment). As predictor variables, we included the UV treatment (non-UV-blocked/UV-blocked), brood-size manipulation (enlarged/reduced), the interaction between both treatments, the day of the video recording (day 12/13), the original brood size and the Julian hatching date. We also included the interactions between UV treatment and all other predictors, which were never significant (see Results). We did not include the interactions between brood size manipulation and the rest of the variables to avoid more than parameterization, and because the brood size was manipulated to observe the effect of UV color on behavioral parameters.

To analyze the individual body mass change, we used a mixed model with normal distribution, which included dyad ID and nest ID (nested within dyad ID) as random factors, and the same two random slopes as above. Moreover, we included a new categorical variable (foster nestling yes/no) as a random factor to control for whether each nestling had been exchanged or not. We included the same predictor variables as in previous models, as well as nestling sex and all the interactions between UV treatment and predictor variables.

## RESULTS

**Figure 1:**
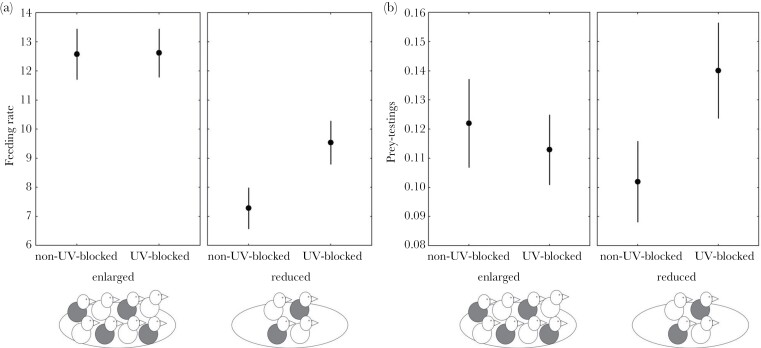
(a) Feeding rate (i.e., total number of prey items which parents delivered during the 30-min observation period) and (b) prey-testings (i.e., log-mean of the number of nestlings which were prey-tested by parents during a feeding bout) according to nestling UV color manipulation (non-UV-blocked/UV-blocked chicks) and brood size manipulation (enlarged/reduced). Values are mean ± SE.

Parental feeding rates were affected by the interaction between both treatments (*P* = 0.0087; Table 1; Figure 1a). Parents preferentially fed UV-blocked nestlings in reduced broods (post hoc test; *t*_1.37_ = –3.43; *P* = 0.008) but had no feeding preferences according to UV treatment in enlarged broods (post hoc test; *t*_1.37_ = 0.16; *P* = 0.99). The number of nestlings which were prey-tested by parents was significantly affected by the interaction between treatments (*P* = 0.0017; Table 1; Figure 1b). Parents prey-tested UV-blocked nestlings significantly more often in reduced broods (post hoc test; *t*_73.3_ = –3.22; *P* = 0.013), whereas there was no difference between nestlings with different treatment in enlarged broods (post hoc test; *t*_73_ = 0.92; *P* = 0.80).

**Table 1:** Mixed models showing the effects of nestling UV treatment (non-UV-blocked/UV-blocked) and brood size manipulation (enlarged/reduced) on the feeding rate (i.e., number of prey items which the nestlings received) provided by blue tit parents, on the prey-testings (i.e., log-mean of the number of nestlings which were prey-tested by parents), on the nestling begging (i.e., mean of the nestling begging intensity) and on the individual body mass change of nestlings. Significant effects (*P* < 0.05) are marked in bold

	Feeding rate	Prey-testings	Nestling begging	Body mass change
**Intercept**	Coef = 1.84 ± 0.51	Coef = –0.22 ± 0.13	Coef = –1.69 ± 0.83	Coef = 0.62 ± 0.47
**Nestling UV treatment** (non-UV-blocked)	Coef = –0.27 ± 0.08 *F*_1,39_ = 6.63 ***P* = 0.014**	Coef = –0.04 ± 0.01 *F*_1,39.1_ = 2.20 *P* = 0.15	Coef = –0.18 ± 0.09 *F*_1,38.6_ = 4.27 ***P* = 0.046**	Coef = –0.04 ± 0.03 *F*_1,672_ = 2.82 *P* = 0.094
**Brood size manipulation** (enlarged)	Coef = 0.28 ± 0.10 *F*_1,37_ = 20.22 ***P* < 0.0001**	Coef = -0.03 ± 0.01 *F*_1,37.1_ = 0.20 *P* = 0.65	Coef = 0.83 ± 0.11 *F*_1,35.2_ = 62.44 ***P* < 0.0001**	Coef = 0.09 ± 0.06 *F*_1,39.5_ = 2.82 *P* = 0.10
**Day of brood size** **manipulation** (day 12)	Coef = 0.03 ± 0.09 *F*_1,37_ = 0.11 *P* = 0.74	Coef = 0.02 ± 0.01 *F*_1,37.6_ = 3.67 *P* = 0.063	Coef = –0.04 ± 0.11 *F*_1,36.3_ = 0.11 *P* = 0.74	Coef = 0.30 ± 0.06 *F*_1,39.5_ = 29.39 ***P* < 0.0001**
**Original brood size**	Coef = 0.12 ± 0.04 *F*_1,37_ = 11.02 ***P* = 0.0020**	Coef = 0.01 ± 0.008 *F*_1,34_ = 2.07 *P* = 0.16	Coef = 0.21 ± 0.05 *F*_1,34.6_ = 14.82 ***P* = 0.0005**	Coef = 0.004 ± 0.03 *F*_1,32.4_ = 0.02 *P* = 0.90
**Hatching date**	Coef = –0.02 ± 0.01 *F*_1,37_ = 3.47 *P* = 0.071	Coef = 0.006 ± 0.002 *F*_1,19.5_ = 5.68 ***P* = 0.028**	Coef = 0.03 ± 0.02 *F*_1,37.1_ = 4.77 ***P* = 0.035**	Coef = -0.01 ± 0.007 *F*_1,15.1_ = 2.31 *P* = 0.15
**Nestling sex** (males)				Coef = 0.04 ± 0.03 *F*_1,688_ = 2.58 *P* = 0.11
**Nestling UV treat.** ^*^ **Brood manip.**	Coef = 0.28 ± 0.10 *F*_1,37_ = 7.68 ***P* = 0.0087**	Coef = 0.05 ± 0.02 *F*_1,38.8_ = 11.30 ***P* = 0.0017**		

**Figure 2:**
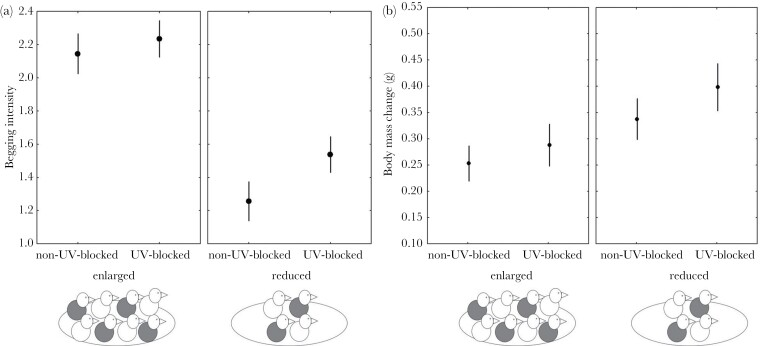
(a) Begging intensity (i.e., mean of the nestling begging intensity based on a five-point rating scale) and (b) individual body mass change (g) of nestlings during 24 h according to nestling UV color manipulation (non-UV-blocked/UV-blocked) and brood size manipulation (enlarged/reduced). Values are mean ± SE.

Overall, UV-blocked nestlings begged more intensely than their non-UV-blocked siblings regardless of brood size manipulation (*P* = 0.046; Table 1; Figure 2a), and nestlings begged more in enlarged broods than in reduced ones (*P* < 0.0001; Table 1; Figure 2a). However, we did not find a significant interaction effect between UV and brood size manipulation on begging intensity (*F*_1,38_ = 2.49; *P* = 0.12). In addition, the larger the original brood size, the higher the begging intensity (*P* = 0.0005; Table 1).

There was no significant interaction effect between treatments on nestling body mass change (*F*_1 672_ = 0.11; *P* = 0.12). Neither was there an effect of UV treatment on nestling body mass change (Table 1; Figure 2b). Moreover, body mass gain was higher between days 11 and 12 (*P* < 0.0001; Table 1), and the effect was independent of the brood size manipulation.

## DISCUSSION

Our results reveal a brood-size mediated parental favoritism for an offspring quality signal (UV color). As predicted, parents preferentially fed the offspring signaling poor quality (UV-blocked) when the parental rearing capacity was not more than challenged (reduced broods). Thus, they followed a brood survival strategy by improving the condition of the weaker offspring. However, in experimentally enlarged broods, which supposedly surpass parental rearing capacities, we expected that parents would follow a brood reduction strategy, by preferentially feeding offspring signaling high quality, but here they did not show parental favoritism.

Whereas parents were expected to actively induce brood reduction, they could have passed the decision more than brood reduction to their offspring by letting them scramble and compete for food. Indeed, it has been suggested that offspring are in a much better position to control parental food allocation in large broods, in which a scramble competition becomes more likely (Royle et al. 2002). Thus, in the current study, sibling competition in enlarged broods could ultimately lead to brood reduction, favoring the survival of the most competitive, high-quality nestlings (Davis et al. 1999; Wright and Leonard 2002; Caro et al. 2016; but see Soler et al. 2022). This could explain why the experimental UV manipulation, which was aimed at changing the perceived individual quality, but not the actual quality itself, did not have an effect on parental provisioning in enlarged nests. So the absence of parental favoritism could, in fact, represent an (indirect) parental brood-reduction strategy which favors the survival of the most competitive siblings (Ricklefs 1993; Jeon 2008). This would be especially relevant in species with facultative asynchronous hatching as the blue tit, with strong age- and size-based hierarchies (Amudsen and Slagsvold 1991; Stenning 1996). Another explanation for our result is that enlarged and reduced broods provide parents with contrasting scenarios of signal perception, which could explain the lack of favoritism. In enlarged broods, parents might have less time to selectively feed given the higher feeding rate. Hence, they might have more difficulties distinguishing UV-blocked and non-UV-blocked treatments, because nestlings would form a tangle of begging nestlings. This did not happen in reduced broods in which parents could probably discriminate better and had more time for their feeding decisions. Nonetheless, this explanation seems less likely, given that nestlings begged more intensely in enlarged broods (Figure 2a) and, thus, yellow breast feathers were inevitably more easily visible for parents in these nests. In any case, our results show that parents adjusted their decisions flexibly and in a context-dependent way about whether to rely on offspring signals and, thus, about whom to feed (Carlisle 1982; Bonsall and Klug 2011).

UV-blocked nestlings were prey-tested more often than their sibs in reduced broods, but again there was no such difference in enlarged ones. Previous studies suggested that parents prey-test UV-blocked chicks more often, although only when the rearing capacity is apparently limited (i.e., less than natural conditions compared with food-supplemented nests; Morales and Velando 2018; García-Campa et al. 2021). However, in the current study, when nestling demand exceeded the rearing capacities (enlarged broods), parents did not modify prey-testings. Only in reduced nests, parents increased prey-testings directed to UV-blocked nestlings, perhaps as a strategy to accurately assess offspring hunger levels and thus to shift their investment towards the offspring in poorer conditions. Parents might initially be more reluctant to feed UV-blocked (low-quality) nestlings without testing them in reduced nests but then re-considered their feeding decision when realizing that chick hunger level was higher than that of non-UV-blocked siblings. Prey-testings have also been suggested as the result of gape-size constraints (Wiebe and Slagsvold 2012). However, nestlings did not differ in body mass gain according to treatment, so gape sizes were likely comparable.

Nestlings begged more intensely in enlarged broods independently of their UV treatment and also in broods which were originally larger, supporting the typical pattern of insufficient food supply and increased sib-sib competition in large broods (Wright and Leonard 2002; Hinde and Godfray 2011; Caro et al. 2016). Also, UV-blocked nestlings begged overall more intensively than their non-UV-blocked siblings (Figure 2a). This rapid change in begging behavior is in line with previous evidence showing that UV-blocked nestlings always beg more regardless of resource availability (Morales and Velando 2018; García-Campa et al. 2021). Higher begging levels typically indicate higher hunger levels (see above; Wright and Leonard 2002; Hinde and Godfray 2011). Intriguingly, however, here we experimentally manipulated UV color, and thus the chicks did not differ in the condition a priori, and we did not find differences in growth (see below). This suggests that other family members might have perceived UV-blocked nestlings as individuals in lower condition and treated them differently, which indirectly affected the behavior of the UV-blocked nestlings, that is, they increased their begging levels. Recently, we also could show that UV color influences behavioral interactions among siblings, supporting the idea which siblings respond to the manipulation and that the changes in the behavior of UV-blocked chicks could result from that (García-Antón et al. 2023), in press. Intriguingly, feeding rates were actually higher for UV-blocked nestlings in reduced nests, which could suggest that UV-blocked nestlings increased begging intensity beyond their own current need as such which they were exploiting their parents’ favoritism in reduced broods. Additionally, both begging intensity and prey-testings were positively related to the hatching date. This could be due to the fact that food availability decreased, as typically occurs when the breeding season progresses (Goodship and Buchanan 2007; Heist and Ritchison 2016). Similarly, parents might be more reluctant to feed nestlings without testing them when resources are more limited.

We did not find a significant effect of UV color manipulation on body mass gain, even though UV-blocked nestlings actually received more food, at least in reduced broods. However, UV-blocked nestlings also received more prey-testings, which perhaps imposed a cost on the chicks and thus resulted in comparable growth patterns in non-UV-blocked siblings. It should also be noted that behavior is the first trait which changes in response to a signal, whereas physiological and morphological traits may follow. Thus, if the downstream effects of UV blocking on body mass are (significantly) smaller than the effects of the brood size manipulation, it is also possible which requires more extensive or longer brood size manipulations to capture differences in body mass between UV treatments.

## CONCLUSIONS

This study provides the first experimental evidence that brood size mediates parental favoritism for an offspring quality signal. Parents favored offspring signaling lower quality when their parental rearing conditions were sufficient to raise all nestlings. On the contrary, they did not rely on the UV signal in enlarged broods, either because it was not relevant to their strategy, or because they let the offspring compete and scramble for food. The latter would also favor the survival of the most competitive and high-quality nestlings. However, both context-dependency and flexibility in the use of offspring signals relate to parental rearing capacities, suggesting that offspring might be unaware of the conditions that mediate parental favoritism and parental control, and hence of the costs and benefits of signaling their own condition. Still, the occurrence of favoritism ultimately depends on the fitness costs, which are driven by the balance between parental rearing capacities and nestling demand.

## Data Availability

Analyses reported in this article can be reproduced using the data provided by García-Campa et al (2023). **Conflict of Interest**: The authors declare no conflict of interest.

## Notes

We thank Lucía Mayor Fidalgo for her help during molecular sexing and Daniel Díaz and Alejandra Mira (from the Centro de Gestión del Parque Regional de la Cuenca Alta del Río Manzanares) for logistic support. We also would like to thank an anonymous reviewer and the associate editor, Per Smiseth, for their valuable comments.
